# Delayed Sentinel Lymph Node Dissection in Patients with a Preoperative Diagnosis of Ductal Cancer In Situ by Preoperative Injection with Superparamagnetic Iron Oxide (SPIO) Nanoparticles: The SentiNot Study

**DOI:** 10.1245/s10434-022-13064-0

**Published:** 2023-01-31

**Authors:** Andreas Karakatsanis, Staffan Eriksson, Lida Pistiolis, Roger Olofsson Bagge, Gyula Nagy, Vivian Man, Ava Kwong, Fredrik Wärnberg, the SentiNot Trialists Group, Imad Mohammed, Abdi Fatah Hersi, Allan Jazrawi, Helena Olofsson, Peter Stålberg

**Affiliations:** 1 Department of Surgical Sciences, Uppsala University, Uppsala, Sweden; 2 Department of Surgery, Uppsala University Hospital, Uppsala, Sweden; 3 Centre for Clinical Research, Uppsala University, Västerås, Sweden; 4 Department of Surgery, Västmanland County Hospital, Västerås, Sweden; 5 Department of Surgery, Sahlgrenska Center for Cancer Research, Institute of Clinical Sciences, University of Gothenburg, Sahlgrenska Academy, Gothenburg, Sweden; 6 Department of Surgery, Sahlgrenska University Hospital, Gothenburg, Sweden; 7 Wallenberg Centre for Molecular and Translational Medicine, University of Gothenburg, Gothenburg, Sweden; 8 Breast Unit, Department of Surgery, Linköping University Hospital, Linköping, Sweden; 9 Department of Surgery, University of Hong Kong, Hong Kong, Hong Kong SAR China; 10 Department of Surgery, The University of Hong, Kong-Shen Zhen Hospital, Shenzhen, China; 11 Department of Surgery, Hong Kong Sanatorium and Hospital, Hong Kong, Hong Kong SAR China

## Abstract

**Background:**

Difficulty in preoperatively assessing the risk for occult invasion or surgery that precludes future accurate axillary mapping in patients with ductal cancer in situ (DCIS) account for overutilization of SLND.

**Methods:**

Prospective, multicenter, cohort study, including women with any DCIS planned for mastectomy or DCIS grade 2 and > 20 mm, any DCIS grade 3, any mass-forming DCIS and any planned surgery. Patients received an interstitial SPIO injection during breast surgery, but no upfront SLND was performed. If invasion was identified on final pathology, delayed SLND (d-SLND) was performed separately with the coadministration of isotope ± blue dye (BD). Study outcomes were proportion of upfront SLNDs that were avoided, detection rates during d-SLND, and impact on healthcare costs.

**Results:**

In total, 78.7% of study participants (*N *= 254, mean age 60 years, mean DCIS size 37.8 mm) avoided upfront SLND. On d-SLND (median 28 days, range 9–46), SPIO outperformed Tc^99^ with (98.2% vs. 63.6%, *p* < 0.001) or without BD (92.7% vs. 50.9%, *p *< 0.001) and had higher nodal detection rate (86.9% vs. 32.3%, *p* < 0.001) and with BD (93.9% vs. 41.4%, *p* < 0.001). Only 27.9% of all SLNs retrieved were concordant for Tc^99^ and SPIO. Type of breast procedure (WLE vs. oncoplastic BCT vs. mastectomy) affected these outcomes and accounted for the low performance of Tc^99^ (*p* < 0.001). d-SLND resulted in a 28.1% total cost containment for women with pure DCIS on final pathology (4190 vs. 5828 USD, *p* < 0.001).

**Conclusions:**

Marking the SLN with SPIO may avoid overtreatment and allow for accurate d-SLND in patients with DCIS.

**Supplementary Information:**

The online version contains supplementary material available at 10.1245/s10434-022-13064-0.

Axillary evaluation in a preoperative diagnosis of ductal cancer in situ (DCIS) is not routine practice, as risk for nodal metastases for pure DCIS is extremely low (0.2–0.7%), mainly due to undiagnosed occult invasion.^1,2^ This is observed in 20–30% of patients, and then sentinel lymph node dissection (SLND) is indicated.^1^

Agreement for upfront SLND exists only in the setting of mastectomy, as its feasibility afterwards is highly debated. Regarding breast-conserving therapy (BCT), SLND as a second procedure is considered feasible and accurate.^2–4^ Guidelines advocating against upfront SLND if BCT is planned were mostly based on literature investigating the feasibility of SLND after previous breast surgery,^3–6^ but when specifically addressing SLND detection in the postoperative period after BCT, it seems that false-negative rates (FNR) are higher.^7^ These results stem from studies with small numbers and after simple wide local excision (WLE) but nonetheless raise a concern, whereas no data for oncoplastic BCT (OPBCT) are currently available.

Superparamagnetic iron oxide (SPIO) nanoparticles are a SLND tracer with comparable performance to the radioisotope (Tc^99^), with the advantage that it can be injected during the preoperative period.^8,9^ Nonmetallic instruments are usually utilized to avoid the interference of the signal, whereas skin staining and magnetic resonance imaging (MRI) artifacts may be avoided by a deeper, peritumoral intraparenchymal injection.^10^ Marking the SLN in a preoperative diagnosis of DCIS by injecting SPIO in the breast in primary surgery was tested in the SentiNot trial to introduce the concept of delayed SLND (d-SLND). An interim analysis demonstrated that unnecessary upfront SLND could be avoided with significant incremental cost containment, whereas SPIO detection was higher.^11^

In the present report, complete trial results are discussed with focus in the accuracy of SLND after DCIS excision. The SentiNot technique provides a unique possibility to define whether the SLNs detected during SLND as a second session after primary breast resection are the true SLNs, because SPIO is injected when the lymphatic outflow to the axilla is intact, whereas Tc^99^ is injected at the second surgery.

## Methods

### Study Design

The study was undertaken in six hospitals (5 in Sweden and 1 in Hong Kong June 1, 2015 to September 16, 2019). Patients with any DCIS planned for mastectomy or patients with DCIS grade 2 and > 20 mm, any DCIS grade 3 or any mass-forming DCIS, regardless of type of surgery were included. At surgery, patients received an interstitial 2-ml injection of SPIO close to the tumour (Magtrace®, Endomag Ltd, Cambridge, UK), followed by 5-min massage. After surgery, the transcutaneous magnetic signal in the axilla detected by the SentiMag® probe (Endomag Ltd, Cambridge, UK) was registered. If no signal was detected, immediate axillary exploration was performed, and the patient was excluded from the study. If specimen pathology showed invasion, d-SLND was performed; Tc^99^ was then injected, with recommended concomitant use of blue dye (BD). After BCT, the injection site for Tc^99^ and BD was defined by local routines. If mastectomy had been performed, Tc^99^ was injected near the scar or in the periareolar area in nipple-sparing mastectomy.

Transcutaneous magnetic and Tc^99^ signals in the axilla were registered. SLND was conducted with the SentiMag® probe. After SLN retrieval, Tc^99^ signal also was registered; thereafter, the axilla was explored for additional radioactive and blue nodes. Intraoperative frozen section was advised to avoid a third operation. If SLND failed, axillary lymph-node dissection or sampling could be performed based on surgeon’s discretion according to preoperative agreement with the patient. SLNs were considered as magnetic or radioactive if they were detected with the respective probe in situ and ex vivo. Nodes with only ex vivo signal < 10 were considered as nonsentinels for the respective tracer to allow for minimisation of bias due to overlapping between methods. Palpable nodes were excised and registered separately. The procedure was completed when the residual in situ axillary signal was < 10% of the maximum *ex vivo* counts. Blue and brown staining were documented and the presence of SPIO nanoparticles in the SLN was confirmed by the pathologist.

### Study Endpoints

Primary study endpoint was the proportion of unnecessary upfront SLNDs that were avoided, defined as the proportion of patients in the cohort that did not undergo SLND at all. Secondary endpoints included detection rate with the SPIO (d-SLND) compared with Tc^99^ (l-SLND) injected after primary surgery and concordance between the two tracers. SPIO is highly concordant with Tc^99^ in detecting the same SLNs in the upfront SLND setting, as shown in previous meta-analyses.^8^ Therefore, concordance of Tc^99^ injected after recent breast excision (l-SLND) with SPIO injected on intact lymphatics might be considered as a surrogate of the false-negative rate yielded by SLND with injection of Tc^99^ after previous breast excision. We sought to assess the impact of type of surgery, DCIS size and location in the breast, addition of BD, and surgeon proficiency on SPIO detection rate.

### Statistical Analyses

Swedish registry data (2014) demonstrated that 20% of patients with a preoperative diagnosis of DCIS will harbour invasion. At the same period, national guidelines led to upfront SLND in 50% of DCIS diagnoses. A sample of 246 patients would allow confirmation that the true proportion of upgrade to IBC is 20% with 5% uncertainty. The SentiNot technique should allow for d-SLND only to those with IBC, which is a reduction from 50 to 20% (relative reduction = 60%), for which the sample size was adequate (*z*-statistic = 11.763, *p* < 0.0001).

All tests were two-sided, and *p*-value was set to 0.05. Results were presented according to the SAMPL guidelines.^12^ Detection rates were calculated per procedure and per node. A procedure was considered concordant if SPIO and Tc^99^ had retrieved at least one concordant node. Nodal concordance was defined as concordant SLNs divided by magnetic SLNs. Inpatient and outpatient care contacts (visits, time of anaesthesia, surgery and postanaesthetic care, number of pathology reports, frozen section) were directly retrieved; subsequently, the costs were calculated and compared with the standard costs of upfront SLND based on a model and on the assumption that SLND added an average of 30 minutes and that standard pathology without frozen section was performed. This model was provided by the health economy service of Uppsala Care.^13^ Means and rates were presented with 95% confidence intervals (CI) or standard deviation (SD) and medians with range. Demographics included only patient age, whereas race and ethnicity were not prospectively registered. Associations were investigated with univariable analyses and included in multivariable regression analysis if *p* < 0.1. The manuscript was prepared according to the Strengthening the Reporting of Observational Studies in Epidemiology (STROBE) statement.^14^ Health economy outcomes are briefly outlined according to the Consolidated Health Economic Evaluation Reporting Standards (CHEERS) Statement.^15^ Analyses were performed with the IBM SPSS Statistics for Windows, Version 28.0 (IBM Corp, Armonk, NY) and Stata Statistical Software, Release 17 (StataCorp LLC, College Station, TX).

## Results

Patient characteristics are summarized in Table 1. Study protocol was not followed in one patient who was treated with mastectomy, where absence of axillary signal at the end of the primary procedure did not prompt immediate axillary exploration; instead d-SLND was performed. After the exclusion of this case due to protocol violation, in 254 remaining patients with a preoperative diagnosis of DCIS, 65 (25.6%; 95% CI 20.3, 31.4) had invasive cancer (median size 8 mm, range 1–120). One patient, aged > 70 year, was diagnosed with an oestrogen receptor-positive, 6-mm, invasive ductal cancer and nine patients with microinvasion; thus, SLND was not clinically indicated in 18.5% of patients (95% CI 9.3, 31.4) with IBC on pathology (*p *= 0.002). Overall, 78.3% (95% CI 72.8, 83.3) of the participants did not undergo SLND; d-SLND was performed in the remaining 55 patients (21.7%; 95% CI 16.7, 27.2).

**Table 1:** Patient characteristics

*N *= 254		Entire cohort	Subgroup with invasive cancer on pathology (*n *= 65)	Correlation with underlying invasion		
				Univariate analysis	Multivariable analysis	
				*p*-value	Odds ratio (95% CI)	*p*-value
Age, yrs (mean, SD)		60.2 (11.1)	61.6 (10.3)	0.243^*^	n.r.	n.r.
DCIS size, mm (mean, SD)		37.8 (26.8)	43.3 (29.8)	0.087^*^	1.01 (0.99,1.02)	0.090
Nuclear Grade (*n*, %)	1	8 (3.2%)	1 (1.6%)	0.697^#^	n.r.	n.r.
	2	84 (33.2%)	23 (37.7%)			
	3	151 (59.7%)	34 (55.7%)			
	Missing	11 (3.9%)	3 (4.9%)			
Symptomatic lesion (*n*, %)		39 (15.4%)	14 (23.0%)	0.069^#^	0.88 (0.33, 2.30)	0.790
Mass-forming lesion (*n*, %)		45 (17.8%)	17 (27.9%)	0.022^#^	1.82 (0.75, 4.42)	0.184
Type of surgery (*n*,%)	BCT	162 (63.8%)	42 (64.6%)	0.879^#^	n.r.	n.r.
	Mastectomy	92 (36.2%)	23 (37.7%)			
**Wide local excision (WLE)**		**78 (30.7%)**	**16 (24.6%)**	0.169^#^	n.r.	n.r.
**OPBCT**		**82 (32.3%)**	**26 (40.6%)**			
**Mastectomy**		**92 (36.2%)**	**23 (37.7%)**			

Bold values indicate statistical significance

*Student’s *t*-test, #Fisher’s exact test

*BCT* breast-conserving therapy, *DCIS* ductal cancer in situ, *n.r.* not relevant, *OPBCT* oncoplastic breast-conserving therapy, *SD* standard deviation

Delayed SLND (d-SLND) was performed a median of 28 days (range 9–46) after the breast procedure. Blue Dye was injected in 42 procedures (76.4%; 95% CI 63.0, 86.8). The isotope was injected in both the previous lumpectomy site and the periareolar area after BCT and at the skin scar for classic and skin-sparing mastectomy or the nipple-areola complex after nipple-sparing mastectomy. The detection rate of SPIO was higher than Tc^99^, both as a sole tracer (92.7% vs. 50.9%, *p *< 0.001) and in combination with BD (98.2% vs. 63.6%, *p *< 0.001; Table 2). Detailed data are provided in Supplement Table 2 (S2). Associations for SLN detection were sought for age, BMI, DCIS size, nuclear grade, mass effect, location in the breast, type of breast procedure, use of SPIO as standard tracer, time from SPIO injection to d-SLND, isotope injection site, and addition of BD during d-SLND. Successful SPIO detection in univariate analyses interacted with use of SPIO as standard tracer (97.6% vs. 76.9%, difference 20.7%, 95% CI − 2.7, 44.1%, *p *= 0.012), but when BD was administered, this effect disappeared (97.6% vs. 100%, difference 2.4%; 95% CI − 2.2, 7.0, *p *= 0.576). The findings were similar for type of breast surgery (100% for WLE and OPBCT and 80% for mastectomy for SPIO only, *p *= 0.036; 100% for WLE and OPBCT and 95% for mastectomy for SPIO+BD, *p* = 0.564). No effects were retained on multivariable regression analysis. Looking into Tc^99^ detection on univariate analyses, the use of BD increased overall detection (63.6% vs. 50.9%, difference 12.7%; 95% CI 2.1, 23.4, *p *= 0.016), whereas the type of breast procedure was a significant predictor of successful detection (WLE 100.0%; OPBCT 33.3%, mastectomy 45.0%; *p *< 0.001 for sole Tc^99^ and WLE 100.0%, OPBCT 58.3%, mastectomy 50.0%; *p *= 0.009 for Tc^99^+BD). The increase of Tc^99^ detection with BD was significant only for OPBCT (58.3% vs. 33.3%, difference 25.0%; 95% CI 3.5, 46.5, *p *= 0.031). In logistic regression, the addition of BD was not significant for Tc^99^ detection (odds ratio 2.053; 95% CI 0.623, 6.767, *p *= 0.237), but type of breast procedure remained significant; OPBCT and mastectomy were associated with less probability for successful Tc^99^ detection (odds ratio 0.332; 95% CI 0.136, 0.810, *p *= 0.015).

**Table 2:** Detection rates per patient in delayed SLND

Detection rate (%)					
	SPIO	Tc^99^	Difference	95% CI	*p*
WLE	100.0	100.0	0.0	− 9.1, 9,1	1.000
OPBCT	100.0	33.3	66.7	43.6, 89.7	< 0.001
Mastectomy	80.0	45.0	35.0	−18.3, 71.8	0.092
Total	92.7	50.9	41.8	24.3, 59.3	< 0.001

	SPIO+BD	Tc^99^+BD	Difference	95% CI	*P*
WLE	100.0	100.0	0.0	− 9.1, 9,1	1.000
OPBCT	100.0	58.3	41.7	17.8, 65.6	0.002
Mastectomy	95.0	50.0	45.0	14.2, 75.8	0.012
Total	98.2	63.6	34.5	19.2, 49.9	< 0.001

*BD* blue dye, *CI* confidence interval, *OPBCT* oncoplastic breast-conserving therapy, *SPIO* superparamagnetic iron oxide nanoparticles, *Tc*^99^ Technetium 99, *WLE* wide local excision

*p* value is two-sided and refers to McNemar’s test for paired proportions

With regards to number of SLNs retrieved, SPIO had a higher nodal detection rate than Tc^99^, yielding more SLNs, both as a sole tracer (86.9% vs. 32.3%, *p *< 0.001) and with the addition of BD (93.9% vs. 41.4%, *p *< 0.001) as shown in Table 3. Detailed data are provided in Supplement Table 3 (S3). The performance of SPIO did not interact with any factors, but Tc^99^ was affected by the use of BD and the type of breast procedure, with the only exception of patients that had undergone simple WLE. In multivariable regression, the addition of BD was not significant (incidence rate ratio [IRR]: 1.675; 95% CI 0.871, 3.223, *p *= 0.122), but type of breast procedure was significant; OPBCT and mastectomy were associated with reduced nodal yield (IRR: 0.549; 95% CI 0.362, 0.834, *p *= 0.005). Six patients (10.9%) had metastases in nine SLNs—all successfully identified by SPIO, whereas Tc^99^ was successful in two patients, detecting one metastatic node in each (detailed data in Supplement Table 4; S4).

**Table 3:** Detection rates per sentinel lymph node in delayed SLND

	Nodal detection rate (%)					SLNs (median, IQR)		
	SPIO	Tc^99^	Difference	95% CI	p*	SPIO	Tc^99^	*p*†
WLE	86.9	82.6	4.3	− 19.0, 27.7	1.000	1 (1, 3)	1 (1, 3)	1.000
OPBCT	90.0	12.5	77.5	58.8, 96.2	< 0.001	1 (1, 2)	0 (0, 1)	< 0.001
Mastectomy	83.3	22.0	61.1	36,2, 86.0	< 0.001	1 (1, 2)	0 (0, 1)	0.021
Total	86.9	32.2	54.5	40.9, 68.2	< 0.001	1 (1, 2)	0 (0, 1)	< 0.001

	Nodal detection rate (%)					SLNs (median, IQR)		
	SPIO+BD	Tc^99^	Difference	95% CI	p*	SPIO+BD	Tc^99^	
WLE	95.7	82.6	13.0	− 9.6, 35.7	0.375	2 (1, 3)	1 (1, 3)	0.250
OPBCT	95.0	32.5	62.5	42.1, 82.9	< 0.001	1 (1, 2)	0 (0, 1)	< 0.001
Mastectomy	94.4	30.6	63.8	42.0, 95.8	< 0.001	1 (1, 2)	0 (0, 1)	0.013
Total	93.9	42.4	51.5	38.5, 64.5	< 0.001	1 (1, 2)	1 (0, 1)	< 0.001

*BD* blue dye, *CI* confidence interval, *IQR* interquartile range, *OPBCT* oncoplastic breast-conserving therapy, *SPIO* superparamagnetic iron oxide nanoparticles, *Tc*^99^ Technetium 99, *WLE* wide local excision

*p-value is two-sided and refers to McNemar’s test for paired proportions, †*p*-value is two-sided and refers to Wilcoxon signed-rank test

The concordance between Tc^99^ and SPIO was overall low, with the exception of patients who had undergone simple WLE (Table 4). Overall, at least one SLN was concordant in 29.1% and all SLNs were concordant in 21.8% (difference 7.3%; 95% CI − 4.4, 19.0, *p *= 0.289). Including the BD, at least one SLN was concordant in 45.5% of cases, a significant increase (difference 16.4%; 95% CI − 4.8, 28.0, *p *= 0.008), but this did not affect the “all SLNs concordance” with the difference between one concordant SLN versus all concordant SLNs being significant (difference 23.6%; 95% CI 9.5, 37.8, *p *= 0.002). The increase of concordance with BD addition was significant only for OPBCT, but not WLE or mastectomy. Looking into all definitions of concordance (at least one SLN, all SLNs, with or without BD), only type of breast procedure was significant from all the input variables (age, DCIS size, grade, location in the breast, time from SPIO injection to delayed SLND, Tc^99^ injection site). The addition of BD increased nodal concordance, but this was significant only after OPBCT (49.4% vs. 31.1%, *p *= 0.031). Therefore, no multivariable analysis was indicated.

**Table 4:** Concordance rates between SPIO and Tc^99^

Breast procedure	All SLNs concordant (*n*, %)	At least one SLN concordant (*n*, %)			
	SPIO and Tc^99^ with or without BD	SPIO and Tc^99^ without BD	SPIO and Tc^99^ with BD	Difference (%, 95% CI)	p*
WLE (*n* = 11)	7 (68.6%)	10 (90.9%)	11 (100.0%)	9.1% (− 16.9, 35.2)	1.000
OPBCT (*n* = 24)	2 (8.3%)	2 (8.3%)	8 (33.3%)	25.0% (3.5, 46.5)	0.031
Mastectomy (*n* = 20)	3 (15.0%)	4 (20.0%)	6 (30.0%)	10% (− 8.1, 28.1)	0.500
Total (*n* = 55)	12 (21.8%)	16 (29.1%)	25 (45.5%)	16.4% (− 4.8, 28.0)	0.008
*p*^†^	< 0.001	< 0.001	< 0.001		

Absolute numbers refer to patients

*BD* blue dye, *CI* confidence interval, *OPBCT* oncoplastic breast-conserving therapy, *SPIO* superparamagnetic iron oxide nanoparticles, *Tc*^99^ Technetium 99, *WLE* wide local excision

*p-value is two-sided and refers to McNemar’s test for paired proportions, denoting whether the difference in concordance with or without the definition of BD is statistically significant, †*p*-value is two-sided and refers to Fisher’s exact test, denoting whether concordance rates vary significantly per type of breast procedure

For women who avoided axillary surgery (78.7% of the cohort), the cost reduction was 28.1% (4,190 vs. 5,828 USD, *p *< 0.001; Table 5). A second procedure was costlier, as expected (8851 vs. 6201 USD, *p *< 0.001), but, still, the SentiNot technique resulted in a 9.5% incremental cost containment in the entire cohort population (5321 vs. 5902 USD, *p *= 0.004) due to the significant number of patients that avoided upfront axillary surgery.

**Table 5:** Impact of SentiNot on incremental costs

	Delayed SLND	Upfront SLND	Mean difference	95% CI of the mean difference	*p*
*(Currency SEK)*					
Entire cohort (*n* = 254)	45,645	50,427	− 4782	− 7,990, − 1,573	0.004
DCIS/mIBC	35,805	49,800	− 13,995	− 16,549, − 11,440	< 0.001
Invasive cancer	75,632	52,986	22,646	14,962, 30,329	< 0.001
*(Currency USD)*					
Entire cohort	5321	5902	− 560	− 935, − 184	0.004
DCIS/mIBC	4190	5828	− 1638	− 1938, − 1339	< 0.001
Invasive cancer	8851	6201	2650	1751, 3550	< 0.001

*CI* confidence interval, *DCIS* ductal cancer in situ, *mIBC* microinvasive breast cancer, *SEK* Swedish crowns (currency), *SLND* sentinel lymph node dissection, *USD* U.S. dollars

*p*-value is two-sided and refers to paired Student’s *t*-test

## Discussion

In the SentiNot trial, marking the SLN with SPIO in patients with a preoperative diagnosis of DCIS resulted in avoiding upfront SLND in 78.3% of included patients. Delayed SLND with SPIO yielded higher detection rates than Tc^99^, regardless of breast surgery performed in the first session and in diverse healthcare settings and populations. This provides a novel technique to allow for the safe avoidance of upfront SLND in a preoperative diagnosis of DCIS, regardless of DCIS features or planned surgery and the morbidity that follows axillary surgery regardless of type of breast procedure.^16–18^

Upfront SLND should not be performed in a preoperative diagnosis of DCIS. However, the risk of undiagnosed underlying invasive cancer or the inability to perform accurate axillary mapping causes uncertainty and may explain the variances in practice. Factors, such as size, nuclear grade, and mass-forming lesions have been related with upgrade to invasive cancer, but these results stem from retrospective studies, and there is no consensus.^19–22^ This is mirrored in the inadequate performance of clinical practice guidelines and the difficulty of practicing physicians to adhere to them.^23^ In real-world data, factors, such as surgeon expertise or center caseload, affect the frequency of upfront SLND in DCIS.^24^

Identifying underlying invasion preoperatively is challenging. Preoperative magnetic resonance imaging (MRI) has not been found reliable to predict invasive disease in prospective evaluation.^25^ Vacuum-assisted biopsies (VAB) lower the rate of postoperative upstaging to invasive cancer to 17–18% in retrospective studies,^26,27^ but, in larger cohorts, the rate of underestimation was as high as 25%.^28^ In the prospective CINNAMOME trial (patients with VAB diagnosis of DCIS, planned for mastectomy, average DCIS extent: 69 mm, Grade 3: 51%), the underestimation rate as high as 39%, and it was suggested that upfront SLND should be performed when mastectomy is planned.^29^ The presence of microinvasion has traditionally been viewed as a risk factor^30^ and prompted upfront SLND, but this view is currently being questioned.^31,32^ In DCIS treated with mastectomy, upfront SLND is routinely performed, as impairment of the anatomy afterwards renders SLND extremely challenging,^2–4,33^ a practice that often extends to risk-reducing mastectomies.^34^ However, is 0.5–4.1%, rising up to 11.6% in case of BRCA mutations,^35–37^ implying that the majority of patients would undergo SLND unnecessarily. In this context, extensive preoperative diagnostic workup mammogram with the addition of ultrasound (US) and MRI^38^ or even intraoperative frozen section of the mastectomy specimen^39^ have been discussed, but without data on cost-effectiveness.

It is true that SLND is feasible after previous BCT, but the view that it is accurate is only supported by retrospective data.^40^ Prospective data demonstrate that SLN detection rate after recent BCT is not optimal. In the GATA study, the overall detection rate was only 85.5% and use of only Tc^99^, negative scintigraphy and reoperation in less than 36 days were predictive factors for SLND failure.^41^ DCIS location in the breast or excision size were not associated with SLND failure, but the size or the use of oncoplastic techniques were not described. In the SentiNot trial, patients with DCIS amenable to standard WLE were recruited only if very high-risk features were present. Study inclusion aimed for larger, high-risk lesions, often mass forming, in challenging locations in the breast, planned for oncoplastic BCT or mastectomy. In the OPBCT subgroup, the isotope and BD had very low detection and concordance rates, a finding that did not correlate with DCIS size, location in the breast, time from SPIO injection to breast surgery, or isotope injection site. This finding is in line with other studies that have shown that more extensive procedures affect successful and accurate SLN detection.^42^ The only exception was women who previously underwent WLE. Specifically, nodal concordance between SPIO and Tc^99^ in the SentiNot trial was markedly low after OPBCT or mastectomy, meaning that when Tc^99^ was successful in detecting a lymph node, it was likely not the sentinel lymph node draining the DCIS/IBC area of the breast. In the setting of upfront SLND, SPIO is highly concordant to Tc^99^, and this is unaffected by injections of the tracers in different sites (peritumoral vs. periareolar) in previous studies and meta-analyses.^8,43–46^ This suggests that what accounts for this discordance and the unexpectedly low performance of Tc^99^ in this setting is the extent of dissection that has been performed during the breast procedure. Earlier lymphoscintigraphy studies have showed that a previous excisional biopsy alters the lymphatic drainage patterns in 14–28% of patients.^47,48^ However, the demonstration of this discordance at a nodal level is a novel finding and, together with the low detection rate of Tc^99^, suggest that injection of Tc^99^ on impaired lymphatic outflow may affect detection outcomes, something illustrated post-OPBCT, where the view that SLND is accurate is a mere extrapolation from standard BCT. By allowing for the identification of the “true” SLN, the SentiNot technique provides a unique niche to assess whether it is the same node that is detected by Tc^99^ injected after the resection and to gain insight on the clinical impact of this. In the present study, Tc^99^ did not detect metastases in two thirds of patients with metastases. However, the numbers are too small for robust conclusions, and this matter warrants further investigation.

## Conclusions

The SentiNot technique offers the alternative of marking the SLN in a simple manner in patients who would otherwise be considered for SLND and remove it afterwards, only if needed. In this manner, upfront SLND may be avoided in all cases that may pose a dilemma, such as in, but not limited to, women older than aged 70 years with early-stage, hormone-positive, erbb2-negative breast cancer, as discussed in the Choosing Wisely recommendations.^49^ In the trial, this tailored approach, with review of final pathology at the postoperative multidisciplinary meeting and consideration of patient preference, allowed for 16.9% of patients with microinvasive/invasive breast cancer to avoid SLND. The implementation of the technique was shown to yield potential for significant impact on national level practice, as shown in the interim analysis, whereas incremental cost containment may be substantial in large-scale implementation.^11^

While the results are promising, the study has certain limitations. It was a feasibility trial, mainly addressed to centers familiar with the technique. Additionally, the study was not powered to support the findings on detection rates and discordance; these findings, although novel, are hypothesis-generating and mandate further investigation. Currently, the multicenter, SentiNot 2.0, randomized, controlled trial (ClinicalTrials.gov Identifier:NCT04722692) is accruing data in different countries to allow for robust results regarding the role of d-SLND and whether it could serve as the new standard of care in individualized breast cancer treatment. At the same time, the concept of a prolonged interval from SPIO injection to surgery in the context of neoadjuvant chemotherapy or endocrine therapy has been showed to be feasible, because SPIO does not migrate with time, as recently shown in the phase 2 “Magnetic-assisted UltraSound-guided Sentinel Lymph-Node Biopsy” (MagUS) trial,^50^ and the maximum timeframe between SPIO injection and successful SLN detection is investigated by our group. In an era that the role of standard axillary surgery is being reevaluated, the flexibility that d-SLND provides may prove valuable to avoid overtreatment and its subsequent complications as well as tailor treatment to meet patient needs while sparing potential long-term morbidity and healthcare resources.

## Supplementary Information

Below is the link to the electronic supplementary material.

Supplementary file1 (DOCX 36 KB)
