# Supplementary material for: Delayed Sentinel Lymph Node Dissection in Patients with a Preoperative Diagnosis of Ductal Cancer In Situ by Preoperative Injection with Superparamagnetic Iron Oxide (SPIO) Nanoparticles: The SentiNot Study
Source: Ann Surg Oncol. 2023 Jan 31;30(7):4064–72. doi: 10.1245/s10434-022-13064-0 (PMC10250503; doi:10.1245/s10434-022-13064-0)
Supplement: Supplementary file 1 — Supplementary file1 (DOCX 36 KB) [file 10434_2022_13064_MOESM1_ESM.docx]

**SentiNot 2.0. Supplementary Tables**

**Supplement Table 1 (S1). Patient characteristics**

Legend, Supplement Table 1. *: Student’s t-test, #: Fisher’s exact test, BCT: breast conserving therapy, DCIS: ductal cancer in situ n.r.: not relevant, OPBCT: Oncoplastic breast conserving therapy. In this supplementary version, the exact type of breast procedures performed are broken down in subgroups

**Supplement Table 2 (S2). Detection rates per patient in delayed SLND**

Legend, Supplement Table 2. BD: Blue Dye, CI: Confidence Intervals, OPBCT: Oncoplastic Breast Conserving Therapy, SPIO: Superparamagnetic Iron Oxide Nanoparticles, Tc^99^: Technetium 99, WLE: Wide Local Excision. In this supplementary version, absolute numbers refer to patients; p-value is 2-sided and refers to McNemar’s test for paired proportions.

**Supplement Table 3 (S3). Detection rates per sentinel lymph node in delayed SLND**

Legend, Supplement Table 3. BD: Blue Dye, CI: Confidence Intervals, iqr: interquartile range, OPBCT: Oncoplastic Breast Conserving Therapy, SPIO: Superparamagnetic Iron Oxide Nanoparticles, Tc^99^: Technetium 99, WLE: Wide Local Excision. In this supplementary version, absolute numbers refer to Sentinel Lymph Nodes; *p-value is 2-sided and refers to McNemar’s test for paired proportions. †p-value is 2-sided and refers to Wilcoxon signed rank test.

**Supplement Table 4 (S4). Outcomes of delayed SLND in patients with SLN metastasis**

Legend, Supplement Table 4: OPBCT: Oncoplastic Breast Conserving Therapy, SLN: Sentinel Lymph Node, SPIO: Superparamagnetic Iron Oxide Nanoparticles, Tc^99^: Technetium 99. Absolute numbers in the columns refer to number of Sentinel Lymph Nodes.

**Supplement Table 1 (S1)**

| **N=254** | | Entire Cohort | Subgroup with invasive cancer on pathology (n=65) | **Correlation with underlying invasion** | | |
| --- | --- | --- | --- | --- | --- | --- |
|  |  |  |  | **Univariate analysis** | **Multivariable analysis** | |
|  |  |  |  | **p-value** | **Odds Ratio**  **(95% CI)** | **p-value** |
| Age, yrs (mean, SD) | | 60.2 (11.1) | 61.6 (10.3) | 0.243^*^ | n.r. | n.r. |
| DCIS size, mm (mean, SD) | | 37.8 (26.8) | 43.3 (29.8) | 0.087^*^ | 1.01 (0.99,1.02) | 0.090 |
| Nuclear Grade (n, %) | 1 | 8 (3.2%) | 1 (1.6%) | 0.697^#^ | n.r. | n.r. |
|  | 2 | 84 (33.2%) | 23 (37.7%) |  |  |  |
|  | 3 | 151 (59.7%) | 34 (55.7%) |  |  |  |
|  | Missing | 11 (3.9%) | 3 (4.9%) |  |  |  |
| Symptomatic lesion (n, %) | | 39 (15.4%) | 14 (23.0%) | 0.069^#^ | 0.88  (0.33, 2.30) | 0.790 |
| Mass-forming lesion (n,%) | | 45 (17.8%) | 17 (27.9%) | 0.022^#^ | 1.82  (0.75, 4.42) | 0.184 |
| Type of Surgery  (n,%) | BCT | 162 (63.8%) | 42 (64.6%) | 0.879^#^ | n.r. | n.r. |
|  | Mastectomy | 92 (36.2%) | 23 (37.7%) |  |  |  |
| **Wide local excision (WLE)** | | **78 (30.7%)** | **16 (24.6%)** | 0.169^#^ | n.r. | n.r. |
| **OPBCT (total)** | | **82 (32.3%)** | **26 (40.6%)** |  |  |  |
| *OPBCT level I (glandular rotations, mastopexies)* | | *43 (16.9%)* | *16 (24.6%)* |  |  |  |
| *OPBCT level II (therapeutic mammaplasties, reductions)* | | *33 (13.0%)* | *9 (13.8%)* |  |  |  |
| *OPBCT with perforator flaps* | | *6 (2.4%)* | *1 (1.5%)* |  |  |  |
| **Mastectomy (total)** | | **92 (36.2%)** | **23 (37.7%)** |  |  |  |
| *Mastectomy without immediate reconstruction* | | *70 (27.6%)* | *19 (31.1%)* |  |  |  |
| *Mastectomy with immediate reconstruction* | | *22 (6.0%)* | *4 (6.6%)* |  |  |  |

**Supplement Table 2 (S2)**

|  | Breast Procedure |  | Successful Tc^99^ detection | | |  | Detection Rate (%) | | | | |
| --- | --- | --- | --- | --- | --- | --- | --- | --- | --- | --- | --- |
|  |  |  | Yes | No | Total |  | SPIO | Tc^99^ | difference | 95% CI | p-value |
| Successful SPIO detection | WLE | Yes | 11 | 0 | 11 |  | 100.0 | 100.0 | 0.0 | -9.1, 9,1 | 1.000 |
|  |  | No | 0 | 0 | 0 |  |  |  |  |  |  |
|  |  | Total | 11 | 0 | 11 |  |  |  |  |  |  |
|  |  |  |  |  |  |  |  |  |  |  |  |
|  | OPBCT | Yes | 8 | 16 | 24 |  | 100.0 | 33.3 | 66.7 | 43.6, 89.7 | <0.001 |
|  |  | No | 0 | 0 | 0 |  |  |  |  |  |  |
|  |  | Total | 8 | 16 | 24 |  |  |  |  |  |  |
|  |  |  |  |  |  |  |  |  |  |  |  |
|  | Mastectomy | Yes | 6 | 10 | 16 |  | 80.0 | 45.0 | 35.0 | -18.3, 71.8 | 0.092 |
|  |  | No | 3 | 1 | 4 |  |  |  |  |  |  |
|  |  | Total | 9 | 11 | 20 |  |  |  |  |  |  |
|  |  |  |  |  |  |  |  |  |  |  |  |
|  | Total | Yes | 25 | 26 | 51 |  | 92.7 | 50.9 | 41.8 | 24.3, 59.3 | <0.001 |
|  |  | No | 3 | 1 | 4 |  |  |  |  |  |  |
|  |  | Total | 28 | 27 | 55 |  |  |  |  |  |  |
|  | | | | | | | | | | | |
|  |  |  | Successful Tc^99^+BD detection | | |  | Detection Rate (%) | | | | |
|  |  |  | Yes | No | Total |  | SPIO+BD | Tc^99^+BD | difference | 95% CI | p-value |
| Successful SPIO+BD detection | WLE | Yes | 11 | 0 | 11 |  | 100.0 | 100.0 | 0.0 | -9.1, 9,1 | 1.000 |
|  |  | No | 0 | 0 | 0 |  |  |  |  |  |  |
|  |  | Total | 11 | 0 | 11 |  |  |  |  |  |  |
|  |  |  |  |  |  |  |  |  |  |  |  |
|  | OPBCT | Yes | 14 | 10 | 24 |  | 100.0 | 58.3 | 41.7 | 17.8, 65.6 | 0.002 |
|  |  | No | 0 | 0 | 0 |  |  |  |  |  |  |
|  |  | Total | 14 | 10 | 24 |  |  |  |  |  |  |
|  |  | | | | |  |  | | | | |
|  | Mastectomy | Yes | 9 | 10 | 19 |  | 95.0 | 50.0 | 45.0 | 14.2, 75.8 | 0.012 |
|  |  | No | 1 | 0 | 1 |  |  |  |  |  |  |
|  |  | Total | 10 | 10 | 20 |  |  |  |  |  |  |
|  |  | | | | |  |  | | | | |
|  | Total | Yes | 34 | 20 | 54 |  | 98.2 | 63.6 | 34.5 | 19.2, 49.9 | <0.001 |
|  |  | No | 1 | 0 | 1 |  |  |  |  |  |  |
|  |  | Total | 35 | 20 | 55 |  |  |  |  |  |  |

**Supplement Table 3 (S3)**

|  | Breast Procedure |  | Successful Tc^99^ detection | | |  | Nodal Detection Rate (%) | | | | |  | SLNs (median, iqr) | | |
| --- | --- | --- | --- | --- | --- | --- | --- | --- | --- | --- | --- | --- | --- | --- | --- |
|  |  |  | Yes | No | Total |  | SPIO | Tc^99^ | difference | 95% CI | p-value* |  | SPIO | Tc^99^ | p-value† |
| Successful SPIO detection | WLE | Yes | 17 | 3 | 20 |  | 86.9 | 82.6 | 4.3 | -19.0, 27.7 | 1.000 |  | 1 (1,3) | 1 (1,3) | 1.000 |
|  |  | No | 2 | 1 | 3 |  |  |  |  |  |  |  |  |  |  |
|  |  | Total | 19 | 4 | 23 |  |  |  |  |  |  |  |  |  |  |
|  |  |  |  |  |  |  |  |  |  |  |  |  |  |  |  |
|  | OPBCT | Yes | 3 | 33 | 36 |  | 90.0 | 12.5 | 77.5 | 58.8, 96.2 | <0.001 |  | 1 (1,2) | 0 (0,1) | <0.001 |
|  |  | No | 2 | 2 | 4 |  |  |  |  |  |  |  |  |  |  |
|  |  | Total | 5 | 35 | 40 |  |  |  |  |  |  |  |  |  |  |
|  |  |  |  |  |  |  |  |  |  |  |  |  |  |  |  |
|  | Mastectomy | Yes | 4 | 26 | 30 |  | 83.3 | 22.0 | 61.1 | 36,2, 86.0 | <0.001 |  | 1 (1,2) | 0 (0,1) | 0.021 |
|  |  | No | 4 | 2 | 6 |  |  |  |  |  |  |  |  |  |  |
|  |  | Total | 8 | 28 | 36 |  |  |  |  |  |  |  |  |  |  |
|  |  |  |  |  |  |  |  |  |  |  |  |  |  |  |  |
|  | Total | Yes | 24 | 62 | 86 |  | 86.9 | 32.2 | 54.5 | 40.9, 68.2 | <0.001 |  | 1 (1,2) | 0 (0,1) | <0.001 |
|  |  | No | 8 | 5 | 13 |  |  |  |  |  |  |  |  |  |  |
|  |  | Total | 32 | 67 | 99 |  |  |  |  |  |  |  |  |  |  |
|  |  |  |  |  |  |  |  |  |  |  |  |  |  |  |  |
|  |  | Successful Tc^99^+BD detection | | | |  | Nodal Detection Rate (%) | | | | |  | SLNs (median, iqr) | | |
|  |  |  | Yes | No | Total |  | SPIO+BD | Tc^99^+BD | difference | 95% CI | p-value* |  | SPIO+BD | Tc^99^+BD | p-value† |
| Successful SPIO+BD detection | WLE | Yes | 18 | 4 | 22 |  | 95.7 | 82.6 | 13.0 | -9.6, 35.7 | 0.375 |  | 2 (1,3) | 1 (1,3) | 0.250 |
|  |  | No | 1 | 0 | 1 |  |  |  |  |  |  |  |  |  |  |
|  |  | Total | 19 | 4 | 23 |  |  |  |  |  |  |  |  |  |  |
|  |  |  |  |  |  |  |  |  |  |  |  |  |  |  |  |
|  | OPBCT | Yes | 11 | 27 | 38 |  | 95.0 | 32.5 | 62.5 | 42.1, 82.9 | <0.001 |  | 1 (1,2) | 0 (0,1) | <0.001 |
|  |  | No | 2 | 0 | 2 |  |  |  |  |  |  |  |  |  |  |
|  |  | Total | 13 | 27 | 40 |  |  |  |  |  |  |  |  |  |  |
|  |  |  |  |  |  |  |  |  |  |  |  |  |  |  |  |
|  |  |  |  |  |  |  |  |  |  |  |  |  |  |  |  |
|  | Mastectomy | Yes | 9 | 25 | 34 |  | 94.4 | 30.6 | 63.8 | 42.0, 95.8 | <0.001 |  | 1 (1,2) | 0 (0,1) | 0.013 |
|  |  | No | 2 | 0 | 2 |  |  |  |  |  |  |  |  |  |  |
|  |  | Total | 11 | 25 | 36 |  |  |  |  |  |  |  |  |  |  |
|  |  |  |  |  |  |  |  |  |  |  |  |  |  |  |  |
|  | Total | Yes | 36 | 57 | 93 |  | 93.9 | 42.4 | 51.5 | 38.5, 64.5 | <0.001 |  | 1 (1,2) | 1 (0,1) | <0.001 |
|  |  | No | 6 | 0 | 6 |  |  |  |  |  |  |  |  |  |  |
|  |  | Total | 42 | 57 | 99 |  |  |  |  |  |  |  |  |  |  |

**Supplement Table 4 (S4)**

|  | Type of surgery | Days between breast procedure and delayed SLND | SPIO SLNs | Tc^99^ SLNs | Concordant SLNs | Metastatic SLNs | Metastatic SLNs detected by SPIO+ | Metastatic SLNs detected by Tc^99^ |
| --- | --- | --- | --- | --- | --- | --- | --- | --- |
|  |  |  |  |  |  |  |  |  |
| 1 | OPBCT (inferior pedicle reduction | 44 | 2 | 0 | 0 | 2 | 2 | 0 |
| 2 | OPBCT (crescent mastopexy) | 28 | 2 | 1 | 1 | 2 | 2 | 1 |
| 3 | Mastectomy | 32 | 1 | 0 | 0 | 1 | 1 | 0 |
| 4 | Mastectomy | 27 | 1 | 0 | 0 | 1 | 1 | 0 |
| 5 | Mastectomy | 16 | 4 | 1 | 1 | 1 | 1 | 0 |
| 6 | Mastectomy | 28 | 2 | 1 | 1 | 2 | 2 | 1 |
